# The effect of peripheral defocus on axial growth and modulation of refractive error in hyperopes

**DOI:** 10.1111/opo.12951

**Published:** 2022-02-21

**Authors:** Ian G Beasley, Leon N Davies, Nicola S Logan

**Affiliations:** ^1^ School of Optometry Aston University Birmingham UK

**Keywords:** axial growth, contact lenses, hyperopia, peripheral defocus, refractive error

## Abstract

**Purpose:**

To establish whether axial growth and refractive error can be modulated in hyperopic children by imposing relative peripheral hyperopic defocus using multifocal soft contact lenses.

**Methods:**

A prospective controlled study with hyperopic participants allocated to a control or test group. Control group participants were corrected with single vision spectacles and changes to axial length and refractive error were followed for 3 years. For the test group, axial growth and post‐cycloplegic refractive error were observed with participants wearing single vision spectacles for the first 6 months of the trial and then corrected with centre‐near multifocal soft contact lenses with a 2.00 D add for 2 years. The central ‘near’ portion of the contact lens corrected distance refractive error while the ‘distance’ portion imposed hyperopic defocus. Participants reverted to single vision spectacles for the final 6 months of the study.

**Results:**

Twenty‐two participants, mean age 11.13 years (SD 1.72) (range 8.33–13.92), completed the trial. Axial length did not change during the first 6 months in either group (*p* = 1.00). Axial growth across the 2‐year intervention period was 0.17 mm (SEM 0.04) (*p* < 0.0005) in the test group *versus* 0.06 mm (SEM 0.07) (*p* = 0.68) in the control group. Axial length was invariant during the final 6 months in either group (*p* = 1.00). Refractive error was stable during the first 6 months in both groups (*p* = 1.00). Refractive error change across the 2‐year intervention period was −0.26 D (SEM 0.14) (*p* = 0.38) in the test group versus −0.01 D (SEM 0.09) (*p* = 1.00) in the control group. Neither the test (*p* = 1.00) nor control (*p* = 0.63) group demonstrated a change in refractive error during the final 6 months.

**Conclusions:**

The rate of axial growth can be accelerated in children with hyperopia using centre‐near multifocal soft contact lenses.

**Key points**

- The rate of eye growth can be accelerated in children with hyperopia by imposing relative peripheral hyperopic defocus using multifocal contact lenses.
- Increasing the rate of eye growth in hyperopes using multifocal contact lenses offers a clinically accessible mechanism to reduce the lifelong impact of hyperopia.
- The ability to accelerate eye growth in hyperopes may reduce the burden of refractive error during childhood and mitigate the risks of associated ocular comorbidities later in life.

## INTRODUCTION

Unlike myopia, and despite the known visual consequences^1^, ^2^, ^3^, ^4^, ^5^ and pathological implications of hyperopia,^6^, ^7^, ^8^, ^9^ there has been inertia to address the modulation of refractive error in hyperopic individuals. Hyperopia occurs as a consequence of insufficient ocular growth and a failure to emmetropise in childhood, with the majority of hyperopic refractive errors resulting from an eye that is too short for its refractive power.^10^ The literature demonstrates the ability both to accelerate and retard axial growth in a range of species by imposing single vision, full‐field, relative hyperopic and myopic defocus.^11^, ^12^, ^13^, ^14^, ^15^ In addition, several studies have demonstrated short‐term changes in axial length and choroidal thickness in response to hyperopic defocus in humans.^16^, ^17^, ^18^, ^19^, ^20^ It seems plausible that these principles could be applied to children with hyperopia.

The progression of myopia and axial growth can be modulated in children and adolescents using soft multifocal or dual‐focus contact lenses.^21^, ^22^, ^23^, ^24^ These contact lenses are designed to correct distance refractive error through the central optic zone, while simultaneously imposing myopic defocus through the outer optic zone. For hyperopes, using centre‐near bifocal soft contact lenses to correct distance refractive error through the central optic zone, while simultaneously imposing hyperopic defocus through the outer optic zone, could provide a stimulus to axial growth, thereby increasing the rate of axial elongation and subsequently reducing refractive error. Previous studies have also examined the effect of imposing relative peripheral hyperopia in animals.^25^, ^26^, ^27^ Hitherto, there has been no attempt to impose relative peripheral hyperopic defocus to modulate refractive error and axial growth in children with hyperopia.

Based upon the evidence from animal models and short‐term laboratory studies in adult humans, the objective of this longitudinal clinical trial was to establish whether axial eye growth and refractive error could be modulated in children and adolescents with hyperopia by imposing relative peripheral hyperopic defocus using soft multifocal contact lenses.

## METHODS

Prior to commencing the research, ethical approval was obtained from both the National Health Service (NHS) Health Research Authority and the Aston University Research Ethics Committee, with the study designed to follow the tenets of the Declaration of Helsinki. Each participant, and their parent or guardian where appropriate, was given detailed information regarding the nature of the study, both verbally and in written form; this allowed informed consent and assent to take place prior to participation. Participants were required to complete a short questionnaire to ensure that they met the inclusion criteria. The programme of research was registered as a clinical trial: ClinicalTrials.gov NCT02686879. Suitable candidates for the study were recruited by displaying notices at the research venues.

**FIGURE 1:**
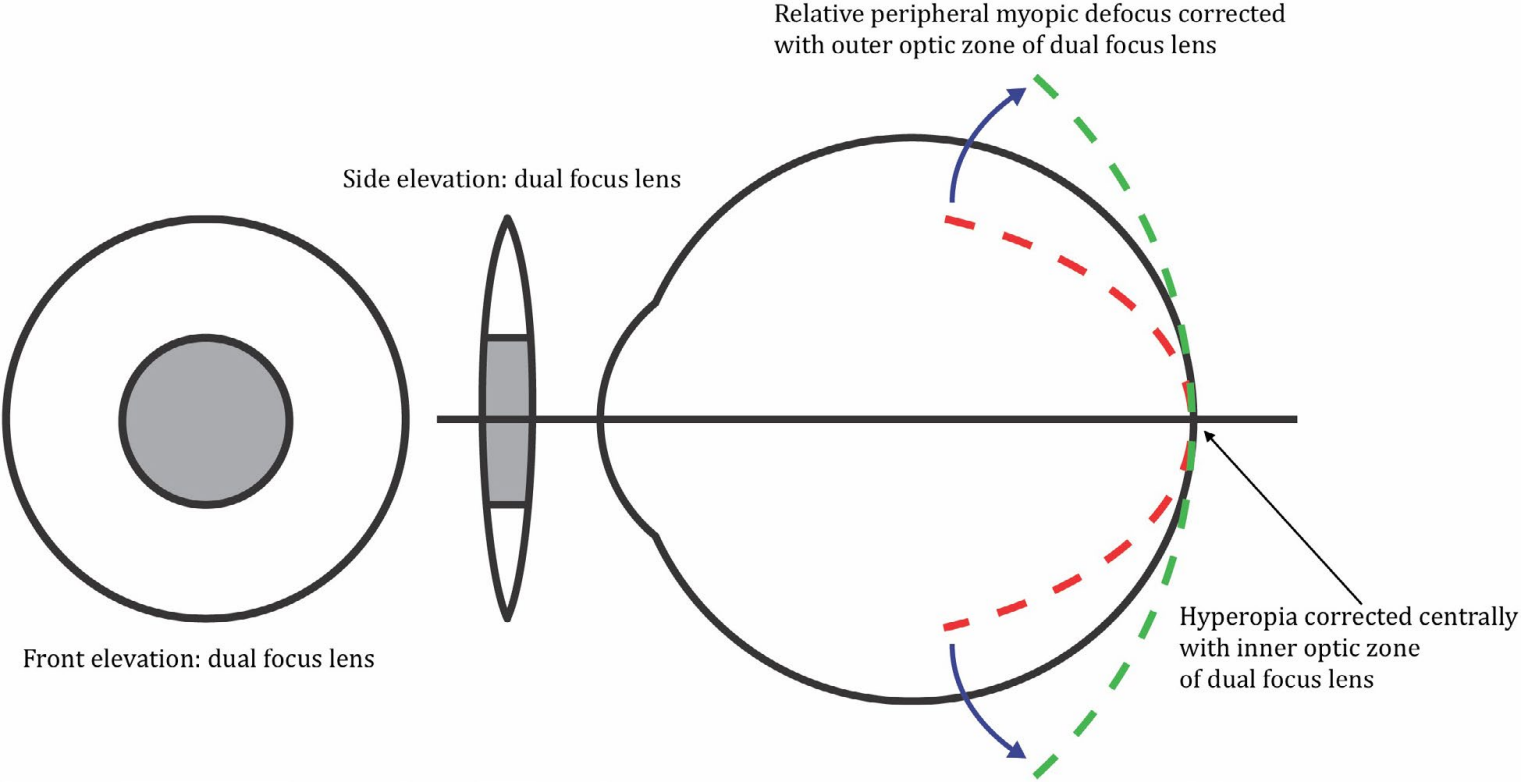
Schematic to demonstrate the concept of relative peripheral hyperopic defocus (RPHD) imposed with a centre‐near bifocal contact lens (CL), while full refractive error is corrected centrally

Participants were allocated to one of two non‐randomised groups:

1. Natural progression group: refractive error and axial growth were followed over a 3‐year period with recruitment open to hyperopes aged between 5 and <20 years‐of‐age to gain an understanding of natural progression of these parameters in the specified cohort. This arm of the study did not involve an intervention, with participants wearing their habitual spectacle correction throughout, and therefore served as a control group for the clinical trial
2. Hyperopic intervention group: for the intervention arm of the trial, axial growth and refractive error were observed without intervention for the first 6 months of the trial with participants wearing their habitual spectacle correction during this period. Between the 6 and 30‐month timepoints of the 3‐year trial, participants wore centre‐near multifocal soft contact lenses bilaterally for a minimum of 10 h per day for 6 days a week. Monthly disposable Biofinity multifocal contact lenses (CooperVision, coopervision.com), with a centre‐near design and an add power of +2.00 D, were worn throughout the intervention period. The power of the central portion of the lens was selected to correct distance refractive error while simultaneously exposing the retina to relative peripheral hyperopic defocus from the outer distance zone (see Figure 1). A +2.00 D add was selected in line with previous refractive error modulation studies^21^, ^22^, ^28^ to strike a balance between ensuring adequate visual performance^29^ while imposing peripheral defocus at a level sufficient to test the hypothesis.^24^ Participants aged between 8 and <16 years‐of‐age were recruited for this arm of the study. For the final 6 months of the trial, the intervention was withdrawn, and participants reverted to optimal spectacle correction, with axial growth and refractive error observed.

Allocation to the respective arms of the study was not randomised. Individuals who were willing and able to use contact lenses were given the opportunity to be included in the contact lens arm of the study in the first instance; those who did not want to wear, were unable to handle or considered unsuitable for contact lenses, were given the opportunity to participate in the natural progression arm of the study. Inclusion and exclusion criteria are summarised in Table 1.

**TABLE 1:** Summary of inclusion and exclusion criteria

Inclusion criteria	Exclusion criteria
Between 5 and <20 years of age for the natural progression group	Previous contact lens wear
Between 8 and <16 years of age for the intervention group	Participating in another clinical study
For participants <16 years‐of‐age, parents must have read, understood and signed the informed consent form	Regular use of medication to treat ocular conditions
Participants must have read, understood and signed the consent or assent form as appropriate	Current use of systemic medication that could impact upon successful contact lens wear or affect focusing ability
Participants in the intervention group agreed to wear contact lenses for a minimum of 10 h per day, 6 days per week for the 2‐year intervention period	Participants who were unable to provide informed consent without the aid of an interpreter due to lack of funding available for the provision of this facility
Be in good general health with no contraindications to contact lens wear	Findings identified during contact lens assessment that would preclude contact lens wear
Maximum manifest spherical refractive error of +6.00 D	Known ocular or systemic disease. Participants with amblyopia/strabismus were not excluded
Maximum manifest cylindrical refractive error of −1.00 D	
Maximum manifest anisometropia of 1.00 D (mean spherical error)	
Minimum manifest mean spherical refractive error of +2.00D in the more hyperopic eye for inclusion in the intervention group	
Be competent at handling contact lenses and understand the instructions given to ensure safe wear	

Measures of unaided distance vision (DV) and distance visual acuity (DVA) at 6 m along with near visual acuity (NVA) at 0.25 m were undertaken with high contrast logMAR charts and determined using a by‐letter scoring method (0.02 logMAR units per letter). Biometric assessment included measures of axial length (AL), anterior chamber depth (ACD) and corneal curvature (CC) and were taken using the IOLMaster 500 (Carl Zeiss Meditec, zeiss.com). For AL, 10 measures were taken per eye and the composite value recorded. Subjective refraction was recorded prior to instillation of cyclopentolate hydrochloride 1% using standard optometric techniques. Measures of accommodative lag were also obtained using the Grand Seiko WAM‐5500 autorefractor (Shin‐Nippon, Rexxam, shin‐nippon.jp). The participant was asked to view a high‐contrast Maltese cross, 25 mm in size, at 0.33 m binocularly while wearing their distance correction, with measures of accommodative lag taken in the dominant eye only,^30^ established using the hole‐in‐the‐card test.^31^ Amplitude of accommodation was assessed using a Royal Air Force (RAF) rule with the mean of 3 push‐up and 3 pull‐down measures reported.^32^, ^33^ Objective central refraction was measured 30 minutes after instillation of the cycloplegic agent using the Grand Seiko WAM‐5500 autorefractor while viewing a diffuse target at 6 m equivalent distance. Post‐cycloplegic peripheral refraction measures were undertaken using the same instrument 30° temporally, 30° nasally, 20° superiorly and 20° inferiorly. Here, participants were asked to fixate on high‐contrast Maltese crosses, 25 mm in size, in photopic conditions (440 lux) which were placed on a wall at 1.64 m to achieve the desired eccentricity points for each of the four peripheral measures. Central contrast sensitivity was recorded with spectacle and contact lens correction using a computerised version of the Pelli‐Robson chart (Thomson Software Solutions, thomson‐software‐solutions.com) at a distance of 1 m. Stereoacuity was measured using the TNO Randot Stereotest (Edition 15, Laméris, lameris‐group.nl) at a distance of 0.4 m with spectacle and contact lens correction.

### Statistical analyses

All data were analysed using the commercially available software SPSS (version 25, IBM, ibm.com). Sample size calculation indicated that 22 participants would be required to achieve 80% power for an effect size of 0.25 at a significance level of 5% using a mixed factor repeated measures analysis of variance (ANOVA) design (G*Power 3.1, Heinrich Heine Universitat, psychologie.hhu.de). Data were examined with Bonferroni correction applied throughout.^34^, ^35^, ^36^ The aim was to recruit 28 participants to allow for an attrition rate of 20%. For the primary outcome measures, the mean longitudinal change in AL was the same for the right and left eyes (*F*_1,10_ = 0.678, *p* = 0.43); this was also the case for post‐cycloplegic refractive error (*F*_1,10_ = 0.281, *p* = 0.61). As such data are presented for the right eye only, which was selected at random.^37^

## RESULTS

**FIGURE 2:**
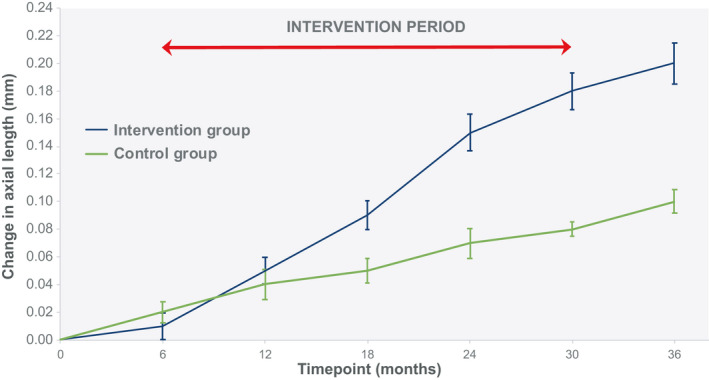
Change in axial length (AL) (mean ± SEM). SEM, standard error of the mean

**FIGURE 3:**
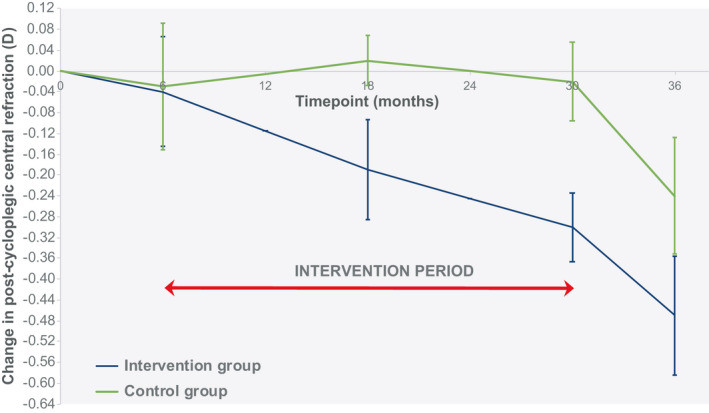
Change in mean spherical equivalent (MSE) post‐cycloplegic, objective, central refraction (mean ± SEM). SEM, standard error of the mean

Twenty‐eight participants were recruited in total, with 16 in the intervention group and 12 in the control group. Due to attrition, 5 participants in the intervention group and 2 participants in the control group did not complete the study, with 1 participant transferring from the intervention group to the control group at the second visit (prior to intervention). There were no adverse events relating to contact lens wear. In total, 22 participants completed the trial with 11 in the intervention group (8 females and 3 males) with an age range at baseline of 8.42–13.5 years, mean 11.13 years (SD 1.72); these data were normally distributed (Z = 0.17, *p* = 0.20). The control group consisted of 11 participants (9 females and 2 males) with an age range of 8.33–13.92 years, mean 11.42 years (SD 2.23); these data were normally distributed (Z = 0.19, *p* = 0.20). The groups were age‐matched (unpaired *t*‐test: *t* = 0.35, df = 20, *p* = 0.73). The data presented here are for participants that completed the full trial. Primary outcome measures are detailed in Tables 2 and 3 and Figures 2 and 3. Secondary outcome measures are summarised in Tables 4 and 5.

**TABLE 2:** Axial length (AL) at each visit

Timepoint (months)	AL (mm)	
Baseline	21.45 (SEM 0.27)	21.81 (SEM 0.27)
6	21.46 (SEM 0.27)	21.83 (SEM 0.27)
12	21.50 (SEM 0.27)	21.85 (SEM 0.28)
18	21.54 (SEM 0.27)	21.86 (SEM 0.27)
24	21.60 (SEM 0.28)	21.88 (SEM 0.28)
30	21.63 (SEM 0.29)	21.89 (SEM 0.28)
36	21.65 (SEM 0.30)	21.91 (SEM 0.28)
	Intervention (*n* = 11)	Control (*n* = 11)

Note

Intervention period shaded orange.

SEM, standard error of the mean.

**TABLE 3:** Mean spherical equivalent (MSE) post‐cycloplegic, objective, central refractive error at each visit

Timepoint (months)	Refractive error (D)	
Baseline	+5.23 (SEM 0.68)	+3.78 (SEM 0.57)
6	+5.19 (SEM 0.67)	+3.75 (SEM 0.56)
18	+5.04 (SEM 0.72)	+3.80 (SEM 0.56)
30	+4.93 (SEM 0.72)	+3.76 (SEM 0.60)
36	+4.76 (SEM 0.69)	+3.54 (SEM 0.59)
	Intervention (*n* = 11)	Control (*n* = 11)

Note

Intervention period shaded orange.

Abbreviation: SEM, standard error of the mean.

**TABLE 4:** Summary of secondary outcomes measures for the test and control groups

Measure	Baseline		6 months		12 months		18 months		24 months		30 months		36 months	
	Test	Control	Test	Control	Test	Control	Test	Control	Test	Control	Test	Control	Test	Control
Unaided DV LogMAR	0.21 (0.10)	0.06 (0.05)	0.22 (0.09) *p* = 1.00	0.06 (0.05) *p* = 1.00	0.22 (0.09) *p* = 1.00	0.04 (0.05) *p* = 1.00	0.21 (0.09) *p* = 1.00	0.04 (0.05) *p* = 1.00	0.20 (0.09) *p* = 1.00	0.03 (0.05) *p* = 1.00	0.21 (0.09) *p* = 1.00	0.04 (0.05) *p* = 1.00	0.20 (0.09) *p* = 1.00	0.04 (0.05) *p* = 1.00
Spectacle DVA LogMAR	0.02 (0.04)	−0.06 (0.03)	0.03 (0.04) *p* = 1.00	−0.07 (0.02) *p* = 1.00	0.00 (0.03) *p* = 1.00	−0.07 (0.03) *p* = 1.00	0.00 (0.03) *p* = 1.00	−0.07 (0.03) *p* = 1.00	−0.02 (0.03) *p* = 1.00	−0.07 (0.03) *p* = 1.00	0.00 (0.04) *p* = 1.00	−0.07 (0.03) *p* = 1.00	−0.01 (0.03) *p* = 1.00	−0.06 (0.03) *p* = 1.00
Spectacle NVA LogMAR	0.23 (0.03)	0.18 (0.03)	0.23 (0.03) *p* = 1.00	0.16 (0.02) *p* = 1.00	0.21 (0.03) *p* = 1.00	0.18 (0.04) *p* = 1.00	0.19 (0.03) *p* = 1.00	0.13 (0.03) *p* = 0.02	0.18 (0.03) *p* = 1.00	0.12 (0.03) *p* = 1.00	0.18 (0.03) *p* = 1.00	0.13 (0.03) *p* = 1.00	0.15 (0.03) *p* = 1.00	0.13 (0.02) *p* = 1.00
Spectacle stereoacuity (sec of arc)	144.00 (41.18)	99.00 (42.44)	132.00 (39.80) *p* = 1.00	102.00 (42.00) *p* = 1.00	150.00 (40.25) *p* = 1.00	111.00 (41.96) *p* = 1.00	126.00 (40.45) *p* = 1.00	90.00 (18.44) *p* = 1.00	113.33 (17.61) *p* = 1.00	98.18 (22.88) *p* = 1.00	133.33 (45.28) *p* = 1.00	130.91 (40.53) *p* = 1.00	126.67 (45.93) *p* = 1.00	136.36 (53.73) *p* = 1.00
Contrast sensitivity with spectacles			1.51 (0.04) *p* = 1.00	1.62 (0.03) *p* = 1.00							1.53 (0.03) *p* = 1.00	1.62 (0.03) *p* = 1.00	1.57 (0.02) *p* = 0.72	1.64 (0.03) *p* = 1.00
CC (mm)	7.80 (0.08)	7.71 (0.10)	7.80 (0.08) *p* = 1.00	7.70 (0.10) *p* = 1.00	7.79 (0.08) *p* = 1.00	7.72 (0.10) *p* = 1.00	7.81 (0.08) *p* = 1.00	7.71 (0.09) *p* = 1.00	7.79 (0.08) *p* = 1.00	7.70 (0.09) *p* = 1.00	7.80 (0.08) *p* = 1.00	7.71 (0.09) *p* = 1.00	7.80 (0.08) *p* = 1.00	7.70 (0.09) *p* = 1.00
ACD (mm)	3.32 (0.11)	3.48 (0.07)	3.32 (0.12) *p* = 1.00	3.47 (0.07) *p* = 1.00			3.34 (0.11) *p* = 0.21	3.48 (0.06) *p* = 1.00			3.35 (0.12) *p* = 1.00	3.49 (0.07) *p* = 1.00	3.36 (0.12) *p* = 1.00	3.50 (0.07) *p* = 0.39
Amplitude of accommodation (D)	11.61 (0.53)	11.61 (0.46)	11.44 (0.51) *p* = 1.00	11.73 (0.38) *p* = 1.00	11.39 (0.50) *p* = 1.00	11.39 (0.27) *p* = 1.00	11.18 (0.41) *p* = 1.00	11.11 (0.39) *p* = 1.00	11.03 (0.37) *p* = 1.00	11.33 (0.44) *p* = 1.00	11.15 (0.43) *p* = 1.00	11.00 (0.36) *p* = 1.00	10.86 (0.34) *p* = 1.00	11.21 (0.36) *p* = 1.00
Accommodative lag with spectacles (D)	0.76 (0.11)	0.82 (0.12)	1.01 (0.10) *p* = 1.00	0.86 (0.14) *p* = 1.00	1.16 (0.08) *p* = 1.00	0.93 (0.09) *p* = 1.00	1.23 (0.09) *p* = 1.00	0.84 (0.10) *p* = 1.00	1.10 (0.05) *p* = 1.00	0.99 (0.10) *p* = 1.00	1.11 (0.06) *p* = 1.00	1.13 (0.09) *p* = 1.00	1.09 (0.10) *p* = 1.00	1.11 (0.07) *p* = 1.00
MSE relative peripheral refraction temporal 30**°** (D)	−2.38 (0.52)	−1.26 (0.37)	−2.62 (0.53) *p* = 1.00	−1.39 (0.33) *p* = 1.00			−2.32 (0.61) *p* = 1.00	−1.26 (0.28) *p* = 1.00			−2.28 (0.59) *p* = 1.00	−0.98 (0.40) *p* = 1.00	−2.29 (0.52) *p* = 1.00	−0.75 (0.40) *p* = 1.00
MSE relative peripheral refraction nasal 30**°** (D)	−0.75 (0.30)	−0.91 (0.27)	−0.51 (0.25) *p* = 1.00	−1.17 (0.28) *p* = 1.00			−0.90 (0.30) *p* = 0.51	−1.30 (0.26) *p* = 1.00			−1.25 (0.52) *p* = 1.00	−1.16 (0.43) *p* = 1.00	−0.77 (0.31) *p* = 1.00	−0.49 (0.22) *p* = 1.00
MSE relative peripheral refraction superior 20**°** (D)	−0.43 (014)	−0.69 (0.22)	−0.71 (0.14) *p* = 1.00	−0.77 (0.22) *p* = 1.00			−1.02 (0.34) *p* = 1.00	−0.81 (0.20) *p* = 1.00			−0.76 (0.14) *p* = 1.00	−0.77 (0.28) *p* = 1.00	−0.58 (0.25) *p* = 1.00	−0.49 (0.19) *p* = 1.00
MSE relative peripheral refraction inferior 20**°** (D)	−0.91 (0.25)	−0.93 (0.30)	−0.57 (0.22) *p* = 1.00	−0.90 (0.31) *p* = 1.00			−0.76 (0.20) *p* = 1.00	−0.95 (0.29) *p* = 1.00			−0.85 (0.22) *p* = 1.00	−0.75 (0.22) *p* = 1.00	−0.66 (0.31) *p* = 1.00	−0.47 (0.22) *p* = 1.00

Note

*p* values are for within‐subject differences between consecutive measures. SEM in brackets. ACD: anterior chamber depth; CC: corneal curvature; DVA: distance visual acuity; MSE: mean spherical equivalent; NVA: Near Visual Acuity

**TABLE 5:** Summary of outcomes comparing spectacle versus contact lens measures for the test group

Measure	6 months		12 months		18 months		24 months		30 months	
	Spectacle	Contact lens	Spectacle	Contact lens	Spectacle	Contact lens	Spectacle	Contact lens	Spectacle	Contact lens
DVA: spectacle *versus* contact lens	0.03 (0.04)	0.16 (0.03)	0.00 (0.03) *p* = 0.91	0.10 (0.03) *p* = 0.26	0.00 (0.03) *p* = 1.00	0.09 (0.02) *p* = 1.00	‐0.02 (0.03) *p* = 1.00	0.08 (0.03) *p* = 1.00	0.00 (0.04) *p* = 1.00	0.09 (0.03) *p* = 1.00
NVA: spectacle *versus* contact lens	0.23 (0.03)	0.26 (0.02)	0.21 (0.03) *p* = 1.00	0.24 (0.02) *p* = 0.82	0.19 (0.03) *p* = 1.00	0.22 (0.03) *p* = 0.02	0.18 (0.03) *p* = 1.00	0.22 (0.02) *p* = 1.00	0.18 (0.03) *p* = 1.00	0.22 (0.02) *p* = 1.00
Stereoacuity: spectacle *versus* contact lens			150.00 (40.25)	192.00 (50.44)			126.00 (20.88) *p* = 0.37	216.00 (49.15) *p* = 0.17		
Contrast sensitivity: spectacle *versus* contact lens	1.51 (0.04)	1.50 (0.03)							1.53 (0.03) *p* = 0.59	1.54 (0.03) *p* = 0.19
Accommodative lag: spectacle *versus* contact lens			1.16 (0.08)	1.48 (0.10)			1.10 (0.05) *p* = 0.48	1.34 (0.18) *p* = 0.59		
Central contact lens power		+3.93 (0.74)		+3.82 (0.73) *p* = 0.16		+3.73 (0.74) *p* = 0.38		+3.57 (0.73) *p* = 0.019		+3.55 (0.74) *p* = 1.00
Peripheral refraction ‐ contact lenses *in situ* temporal 30**°** (D)				+1.91 (0.60)						
Peripheral refraction ‐ contact lenses *in situ* nasal 30**°** (D)				+1.90 (0.33)						
Peripheral refraction ‐ contact lenses *in situ* superior 20**°** (D)				+0.33 (0.35)						
Peripheral refraction ‐ contact lenses *in situ* inferior 20**°** (D)				+0.46 (0.36)						

P values are for within‐subject differences between consecutive measures. SEM in brackets. DVA: distance visual acuity; NVA: near visual acuity

The primary outcome measures were changes to AL and post‐cycloplegic central refractive error.

Overall, AL increased over time (*F*_(6, 120)_ = 27.09, *p* < 0.0001), although an interaction between factors demonstrated this occurred in the intervention group only (*F*_(6, 120)_ = 4.66, *p* < 0.0001). For the intervention group, AL did not change during the first 6 months prior to contact lens wear (*p* = 1.00). Axial growth accelerated throughout the 2 years of intervention (*P* = <0.0001) but did not change once the intervention was withdrawn for the final 6 months of the trial. For the control group, AL did not change across the 3‐year period (*p* = 0.47). Observed power was 0.99. AL data from baseline to the end point of the trial is detailed in Table 2, with changes over time illustrated in Figure 2.

Post‐cycloplegic mean spherical equivalent (MSE) central refractive error decreased over time (*F*_(4, 80)_ = 6.57, *p *< 0.0001) by a similar amount in both the intervention and control groups (*F*_(4, 80)_ = 1.46, *p* = 0.22) and the observed power was 0.44. Refractive error data from baseline to the end point of the trial is given in Table 3, with changes over time provided in Figure 3.

## DISCUSSION

This clinical trial has shown for the first time that the imposition of relative peripheral hyperopic defocus, using multifocal contact lenses, can accelerate axial growth in children with hyperopia. Participants in the control arm of the study demonstrated axial growth rates that were similar to other longitudinal observations in UK children with hyperopia.^38^ Importantly, the axial growth rates seen in controls were significantly outpaced by participants receiving the intervention over the 2‐year period of wearing multifocal contact lenses. In the 6 months prior to receiving the intervention, axial growth rates for the control and intervention groups were the same. Similarly, once contact lens wear ceased during the final 6 months of the trial, the faster growth rates experienced by the intervention group during the 2‐year period of wearing contact lenses reverted to a pace that matched the control group.

As participants in the intervention arm of the trial experienced a faster rate of axial growth than controls, it would be expected that a decrease in hyperopia would also occur. However, although the mean values showed a greater reduction in post‐cycloplegic refractive error in those receiving the intervention compared to the control group, this did not reach a level of significance nor achieve adequate statistical power. Nevertheless, the mean reduction in refractive error for participants receiving the intervention was almost double the decrease in the control group at 0.47 D and 0.24 D, respectively, which offers optimism for further research in this area. Furthermore, the variability observed with the measurement of refraction^39^ may be a factor that compounds the lack of significance.

Although stereoacuity with spectacles was similar for both groups and did not change over time, in the intervention group it was significantly poorer with contact lens correction compared to spectacles, and failed to improve during the 2‐year period of contact lens wear; this is in contrast to findings from earlier work demonstrating that stereoacuity appears to be preserved in multifocal contact lens wear compared to single vision correction, albeit in a presbyopic cohort.^40^ At baseline, contrast sensitivity was better in the control group than the intervention group and did not change over time. Encouragingly, contrast sensitivity was similar with both spectacle and contact lens correction. Overall, participants appeared to adapt well to the novel form of visual correction; in future work it would be worthwhile considering a qualitative evaluation of this outcome.

For measures of anterior eye parameters, as with previous refractive error modulation work in myopes,^23^, ^41^ CC did not change over time in either group. ACD changed over time in both the control and intervention groups by a comparable amount; this suggests that the greater AL change observed in the intervention group is attributable primarily to vitreous chamber depth (VCD) growth.

Accommodative lag was greater with contact lens correction than spectacle correction, which may reflect that hyperopes would be expected to converge less through the former. Given that accommodative lag has been implicated in myopia progression,^42^, ^43^ it is plausible that lag may be a factor in driving axial growth in contact lens‐wearing participants in the present study.

In line with previous work, peripheral refraction was relatively myopic in all four quadrants.^44^, ^45^, ^46^, ^47^, ^48^, ^49^ Importantly, while wearing the intervention, relative peripheral refraction was hyperopic in all four quadrants, demonstrating merit in using centre‐near contact lenses to expose the peripheral retina to hyperopic defocus. The findings observed in the intervention group offers credibility to the hypothesis that exposing the hyperopic eye to relative peripheral hyperopic defocus may provide the necessary signal to stimulate axial growth in children.

Participants in the intervention arm of the study proved to be adept at handling and maintaining their contact lenses, which is in keeping with earlier work on the safety of contact lenses in children.^50^, ^51^ Compliance with wear also appeared to meet the trial objectives, although this was a self‐reported outcome.

In future work, it would be important to establish if a greater effect can be achieved through earlier intervention. Exposing the hyperopic eye to peripheral hyperopic defocus at a younger age, when natural growth is faster, may yield better results as well as potentially improving the visual outcome, for instance, measures of VA and stereopsis. Nevertheless, it is important to recognise the difficulties of recruiting hyperopes, particularly at a young age. Given the nature of refractive error distribution, hyperopes are relatively scarce in comparison to their myopic counterparts. Furthermore, in the absence of amblyopia, low to moderate hyperopes are likely to ‘pass’ rudimentary vision screening at school,^52^ and may not present for routine assessment within primary care optometry due to being able to accommodate to overcome their refractive error. In the UK, those who fail vision screening are typically diverted to secondary care for several years making recruitment at a young age more difficult in a primary care setting. Additional opportunities to expand upon refractive error modulation in children with hyperopia could be through nuanced optical approaches based upon the mechanism established here. In the present trial, a single dose approach was taken using a 2.00 D add in an ‘off‐the‐shelf’ design intended for presbyopic correction. Further work should assess the impact of using different sized central zones and higher add powers to expose the peripheral retina to a greater degree of hyperopic defocus. In addition, as an extension to the present work, it would be interesting to understand if the response to the intervention is related to the magnitude of the baseline refractive error.

In terms of the main objective, being able to demonstrate the impact of the intervention on axial growth in hyperopes is encouraging. However, it is important to recognise the limitation of this work in terms of refractive error outcome, which lacked statistical power and did not reach a significant level.

Having identified a mechanism to modulate axial growth holds promise for hyperopes, and provides a platform for an extension to this work. The ability to accelerate axial growth in hyperopes may help to decrease the risk of ocular comorbidities associated with small globes^6^, ^7^, ^8^, ^9^ as well as reduce the burden of refractive error in these children.^1^, ^2^, ^3^, ^4^, ^5^ Furthermore, multifocal contact lenses in children with hyperopia appear to be well tolerated both from a handling and wearing perspective as well as providing good visual performance.

## CONFLICT OF INTEREST

The authors confirm that they do not have any commercial interest relevant to the subject of the paper. The contact lenses in the study were supplied free of charge by CooperVision. CooperVision did not sponsor the research and does not support or have an opinion regarding any of the content in the paper.

## AUTHOR CONTRIBUTION

**Ian G Beasley:** Conceptualization (equal); Data curation (lead); Formal analysis (lead); Investigation (lead); Methodology (equal); Project administration (lead); Writing – original draft (lead). **Leon N Davies:** Conceptualization (supporting); Data curation (supporting); Formal analysis (supporting); Investigation (supporting); Methodology (supporting); Supervision (equal); Writing – review & editing (equal). **Nicola S Logan:** Conceptualization (equal); Data curation (supporting); Formal analysis (supporting); Funding acquisition (lead); Investigation (supporting); Methodology (equal); Project administration (supporting); Supervision (lead); Writing – original draft (supporting); Writing – review & editing (equal).
